# Ammonia-Assisted Quadrupled-Yield ZIF-67 Derivation Enables Single Oxygen-Dominated Nonradical Oxidation for Enhanced Ciprofloxacin Degradation

**DOI:** 10.3390/ma18184337

**Published:** 2025-09-16

**Authors:** Xiaoxian Hu, Di Zhang, Xinyu Li, Junfeng Wu, Xiang Guo, Hongbin Gao, Minghui Hao, Yingchun Wang, Bang Li, Xinhai Zhang

**Affiliations:** 1 Henan Key Laboratory of Water Pollution Control and Rehabilitation, Henan University of Urban Construction, Pingdingshan 467000, China; 20221057@huuc.edu.cn (X.H.); zhangd08102001@163.com (D.Z.); 20221024@huuc.edu.cn (X.G.); 20171004@huuc.edu.cn (H.G.); minghuihao@yeah.net (M.H.); 20221015@huuc.edu.cn (Y.W.); 20221067@huuc.edu.cn (B.L.); 2 College of Ecology and Environment, North China University of Water Resources and Electric Power, Zhengzhou 450046, China; 3 College of Food and Chemical Engineer, Hebi Polytechnic, Hebi 458030, China; goldhai7527@163.com

**Keywords:** ZIF-67 derivative, ammonia-assisted synthesis, peroxydisulfate activation, ciprofloxacin degradation

## Abstract

The widespread contamination of aquatic systems by ciprofloxacin (CIP)—a persistent fluoroquinolone antibiotic—poses severe ecological risks due to its antibacterial resistance induction. Conventional sulfate radical-based advanced oxidation processes (SR-AOPs) suffer from inefficient catalyst synthesis, exemplified by low-yield ZIF-67 precursors (typically <25%). To address this, a nitrogen-doped carbon composite Co_3_O_4_/N@C was synthesized via ammonia-assisted ligand exchange followed by pyrolysis, using N-doped ZIF-67 as a self-sacrificial template. The ammonia incorporation quadrupled precursor yield compared to ammonia-free methods. This catalyst activated peroxydisulfate (PDS) to degrade 95% CIP within 90 min under the optimized conditions (0.5 g/L catalyst, 2 mmol/L PDS, pH 5), representing a 30% enhancement over non-ammonia analogs. Mechanistic studies identified singlet oxygen (^1^O_2_) as the dominant reactive species, facilitated by N-doped carbon-mediated electron transfer. This strategy overcomes the scalability barrier of MOF-derived catalysts for practical antibiotic wastewater remediation.

## 1. Introduction

The persistence of antibiotics in aquatic environments has raised significant ecological and public health concerns, particularly due to their role in promoting bacterial resistance [1]. Ciprofloxacin (CIP), a second-generation fluoroquinolone antibiotic, is notably recalcitrant owing to its stable heterocyclic structure, which impedes biodegradation [2]. Conventional wastewater treatment processes exhibit limited efficacy (<30% CIP removal) [3], necessitating advanced remediation strategies.

In recent years, common strategies for addressing CIP contamination have included physical [4], biological [5], and chemical treatment technologies [6]. However, physical methods often produce secondary solid waste, raising concerns about potential solid pollution. Meanwhile, biological degradation processes generally require extended treatment times and involve complex system setups [7]. Consequently, there is a pressing need to develop more efficient and environmentally sustainable methods for removing CIP from water. Currently, various advanced treatment technologies, such as adsorption, ozonation, photocatalysis, and persulfate-based oxidation have been successfully employed for CIP removal [8,9,10,11,12,13,14]. Among these, sulfate radical-based advanced oxidation processes (SR-AOPs) has garnered significant attention owing to its broad operational pH range, high stability, and operational simplicity [15]. SR-AOPs represent a viable strategy for leveraging persulfate activation to generate reactive oxygen species (ROS) [16,17,18]. PDS has emerged as a preferred oxidant in SR-AOPs due to its low cost, stability, and ease of handling. Its activation generates diverse ROS, enabling adaptable degradation pathways [19,20]. Activation strategies span homogeneous (e.g., UV, gamma radiation [21], thermal [22], alkaline [23], or transition metal ions [24]) and heterogeneous approaches (e.g., metal oxides or carbon-based materials [25]). Among these, cobalt-based catalysts exhibit exceptional potential for persulfate activation [26,27]. Li et al. [28] constructed cobalt-based catalysts with different morphologies and architectures, and applied them to activate peroxymonosulfate (PMS) for the degradation of tetracycline (TC) and bisphenol A (BPA). The multishelled Co_3_O_4_ nanospheres exhibited removal rates as high as 96.3% for TC and 98.5% for BPA, respectively, while demonstrating excellent recycling performance. Zhou et al. [29] prepared biochar-supported cobalt-based catalysts (Co_3_O_4_@BCC) for activating PMS, which achieved a 99.0% degradation efficiency of phenacetin. The BCC not only facilitated the dispersion of Co_3_O_4_ nanoparticles and enhanced the stability of the catalyst but also provided abundant electron-rich functional groups, which promoted PMS activation and the generation of ROS. Although traditional cobalt-based materials exhibit good catalytic activity, direct exposure to bulk solutions inevitably leads to metal leaching, resulting in secondary pollution. Therefore, it is very important to develop Co_3_O_4_-based materials with a high specific surface area, activity, and stability [30].

The integration of transition metal oxides with carbonaceous materials not only mitigates metal leaching but also enhances catalytic activity [31]. Metal–organic frameworks (MOFs), particularly zeolitic imidazolate frameworks (ZIFs), serve as excellent templates for deriving carbon-based or metal/carbon composites with well-defined morphology, high metal dispersion, and controllable nanostructures, owing to their high surface area, tunable porosity, and atomically dispersed metal sites [32,33] ZIF-67, a cobalt-centered, exhibits superior thermal/chemical stability and cobalt dispersion, minimizing ion leaching [34]. However, its practical application is hindered by low synthesis yields (<25%), which escalate costs and complicate scale-up [35].

In this study, an ammonia-assisted room-temperature synthesis method was employed to prepare ZIF-67, which significantly increased its yield. The ZIF-67 as a precursor, nitrogen-doped cobalt oxide/carbon composites (Co_3_O_4_/N@C) were fabricated through a one-step pyrolysis approach. The Co_3_O_4_/N@C composite was applied to activate PDS for the degradation of CIP, and the mechanism of PDS activation was elucidated. Furthermore, the process parameters for the catalytic degradation of CIP by Co_3_O_4_/N@C were optimized. This yield-enhanced MOF-derivation approach offers a scalable strategy for efficient antibiotic wastewater remediation.

## 2. Materials and Methods

### 2.1. Chemicals and Materials

Ciprofloxacin (CIP), furfuryl alcohol (FFA), tert-butyl alcohol (TBA), p-benzoquinone (BQ), methanol (MeOH), ethanol (EtOH), sulfuric acid (H_2_SO_4_), 2-methylimidazole (2-MI), sodium hydroxide (NaOH), cobalt nitrate hexahydrate (Co(NO_3_)_2_·6H_2_O), sodium persulfate (PDS), ammonium hydroxide (NH_4_OH), humic acid (HA), sodium chloride(Na_2_CO_3_), sodium carbonate(NaCl), sulfadiazine (SDZ), tetracycline hydrochloride (TCH), 4-amino-2,2,6,6-tetramethylpiperidine (TEMP) and 5,5-dimethyl-1-pyrroline N-oxide (DMPO) were commercially obtained from Shanghai Macklin Biochemical Co., Ltd. (Shanghai, China). All supplementary chemicals, acquired at analytical-grade specifications, were utilized without additional processing.

### 2.2. Preparation of Materials

In order to prepare ZIF-67/N crystals, metal and ligand solutions were prepared by dissolving 6 mmol Co(NO_3_)_2_·6H_2_O and 48 mmol 2-MI in 60 mL and 40 mL EtOH, respectively, and 5 mL NH_4_OH was introduced into the ligand solution. Then, under the condition of magnetic stirring, the metal solution was gradually introduced into the ligand solution, and the solution became purple. Constant agitation of the mixture at ambient temperature (25 ± 1 °C) for 2 h preceded a 12 h quiescent aging phase, culminating in the formation of purple precipitate. Following separation via centrifugation at 8000 rpm for 5 min, the purple precipitate was subjected to successive EtOH rinses and then oven-dried at 60 °C for 12 h to obtain a purple solid powder ZIF-67/N [36].

**Figure 1:**
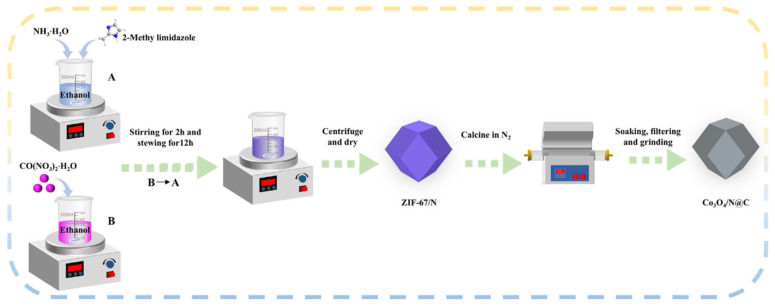
Scheme of the synthesis pathway for Co_3_O_4_/N@C catalysts.

To dehydrate and remove volatile impurities, ZIF-67/N was placed in a crucible and subjected to pyrolysis in a tube furnace. The material was heated under a nitrogen atmosphere at a heating rate of 5 °C·min^−1^ and maintained at different target temperatures (300 °C, 500 °C, and 700 °C) for 2 h, resulting in a black powdered product. The obtained product was immersed in pure water for 12 h to eliminate large metallic particulate matter deposited on the surface of the carbon material. Finally, it was filtered with distilled water 3 or 4 times and oven-dried at 60 °C for 12 h. The black sample was named Co_3_O_4_/N@C according to the precursor (Figure 1).

### 2.3. Characterization of Materials

The morphology and microstructure of the ZIF-67/N and Co_3_O_4_/N@C were characterized using a combination of field-emission scanning electron microscopy (SEM, TESCAN-MIRA4, Brno, Czech Republic) and transmission electron microscopy (TEM, JEOL-JEM-F200, JEOL, Tokyo, Japan). SEM analysis revealed the overall particle shape and size distribution, while TEM provided detailed insights into the lattice fringes and potential graphitic domains within the carbonized samples, which are critical for understanding their catalytic activity in persulfate activation. Powder X-ray diffraction (XRD, Bruker-D8 DISCOVER, Bruker, Karlsruhe, Germany) was employed to analyze the material structure. The composition of the material was further analyzed using X-ray photoelectron spectroscopy (XPS, Thermo Fisher-ESCALAB 250Xi, Thermo Fisher, Waltham, MA, USA). The Raman spectrometer (Thermo Fisher-DXR2xi, Thermo Fisher, Waltham, MA, USA), employing a 532 nm excitation source at room temperature, was utilized to characterize the crystalline carbon structure. Fourier transform infrared spectroscopy (FTIR, Thermo Fisher-Nicole iS 5, Thermo Fisher, Waltham, MA, USA) (scanning range: 400–4000 cm^−1^) confirmed the functional groups present in the catalyst. The magnetic properties of the materials were characterized using a Vibrating Sample Magnetometer (VSM, Lake Shore-8604, Lake Shore Cryotronics, Westerville, OH, USA) at room temperature. The specific surface area and porosity of the catalytic material was determined via surface area measurement method (BET, Micromeritics-ASAP 2460, Micromeritics Instrument Corporation, Norcross, GA, USA). To identify the primary ROS generated during the reaction, electron paramagnetic resonance (EPR, Bruker-A300, Bruker, Karlsruhe, Germany) was employed. Specifically, sulfate radicals (SO_4_·^−^) and hydroxyl radicals (·OH) were detected using DMPO as the spin trap, while singlet oxygen was monitored using TEMP.

### 2.4. Degradation of CIP by Activating PDS and Degradation Efficiency Calculation

All degradation experiments were conducted in 100 mL glass beakers containing 50 mL aliquots of CIP solution at predetermined initial concentrations. Co_3_O_4_/N@C was added to CIP suspension to start the adsorption process. After 30 min, PDS was introduced into the CIP suspension, initiating the degradation process. Throughout the continuous mechanical stirring process, 1 mL aliquots were withdrawn at defined intervals and immediately filtered using a 0.22 μm syringe membrane filter. Furthermore, catalysts synthesized at different carbonization temperatures (e.g., 300 °C, 500 °C, and 700 °C) were compared in parallel degradation tests to determine the optimal carbonization temperature. To optimize CIP degradation efficiency, a systematic investigation was conducted by varying the catalyst loading (0.2, 0.5, 0.8, and 1.0 g/L) and PDS dosage (1, 2, and 3 mmol/L) to identify the optimal combination. Systematic pH variation (3, 5, 7, 9, 10, 11) via H_2_SO_4_ or NaOH addition was employed to assess its impact on CIP degradation efficiency.

CIP concentration was quantified using a high-performance liquid chromatography (HPLC, Thermo Fisher-UltiMate 3000, Thermo Fisher, Waltham, MA, USA) system equipped with a C18 column. The mobile phase comprised acetonitrile and 0.1% acetic acid (3:1, *v*/*v*), delivered at 0.1 mL/min. Detection was performed at 277 nm with the column maintained at 30 °C, employing a 20 μL injection volume. All experiments were repeated three times, and the results were averaged.

## 3. Results and Discussion

### 3.1. Characterization

**Figure 2:**
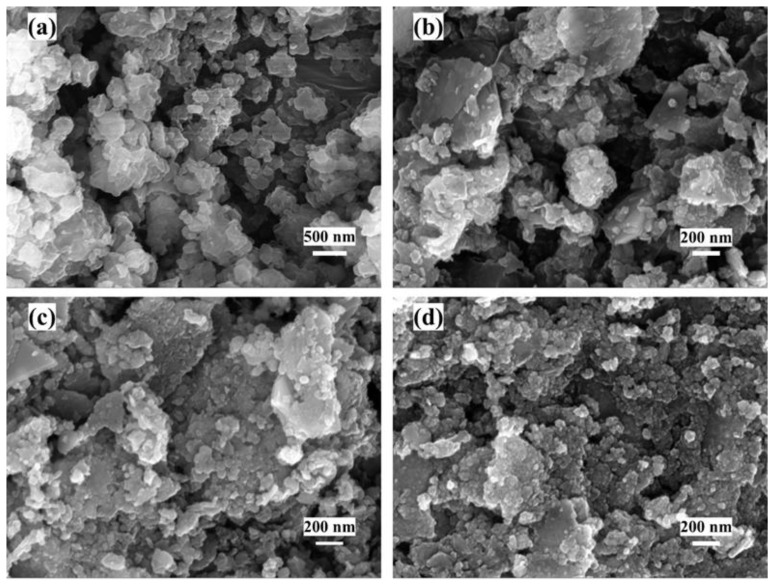
(**a**) SEM images of ZIF-67/N; (**b**) Co_3_O_4_/N@C-300, (**c**) Co_3_O_4_/N@C-500, (**d**) Co_3_O_4_/N@C-700.

The SEM (Figure 2) analysis revealed significant pyrolysis-temperature-dependent morphological evolution. ZIF-67/N (Figure 2a) precursors maintained uniform rhombic dodecahedrons (~200 nm) with smooth surfaces. Pyrolysis at 300 °C induced partial framework collapse and surface wrinkling in Co_3_O_4_/N@C-300 (Figure 2b). Co_3_O_4_/N@C-500 (Figure 2c) developed embedded nanoparticles (20–50 nm) within porous carbon matrices featuring interconnected channels. At 700 °C, Co_3_O_4_/N@C-700 (Figure 2d) exhibited severe sintering with >200 nm aggregates and collapsed porosity, confirming thermal degradation thresholds.

**Figure 3:**
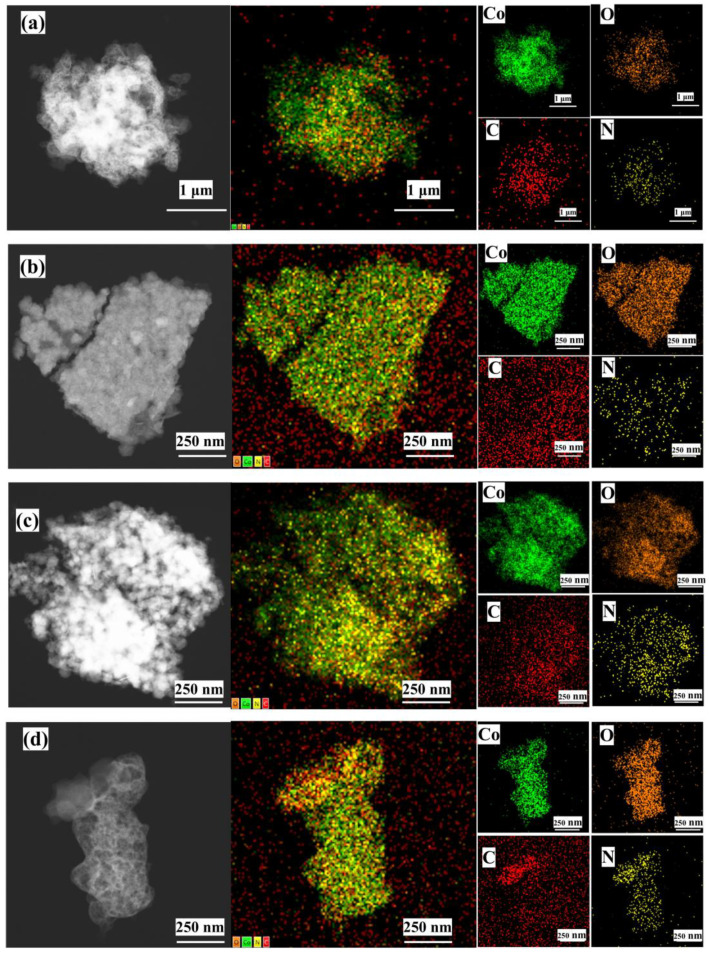
EDS mapping images of the as-prepared Co_3_O_4_/N@C composites calcined at various temperatures. (**a**) ZIF-67/N; (**b**) Co_3_O_4_/N@C-300; (**c**) Co_3_O_4_/N@C-500; (**d**) Co_3_O_4_/N@C-700.

EDS mapping confirmed homogeneous distribution of C, N, O, and Co throughout all Co_3_O_4_/N@C composites (Figure 3). Progressive calcination temperature elevation from 300 °C to 700 °C enhanced the dispersion uniformity of Co and O species, indicating temperature-modulated refinement of metal oxide nanoparticle distribution. Concurrent carbon surface enrichment was evidenced by intensified C signals at higher temperatures. Spatial correlation between O and C elemental maps confirmed oxygen-functionalized carbon matrices, particularly prominent in Co_3_O_4_/N@C-500 and Co_3_O_4_/N@C-700 samples.

**Figure 4:**
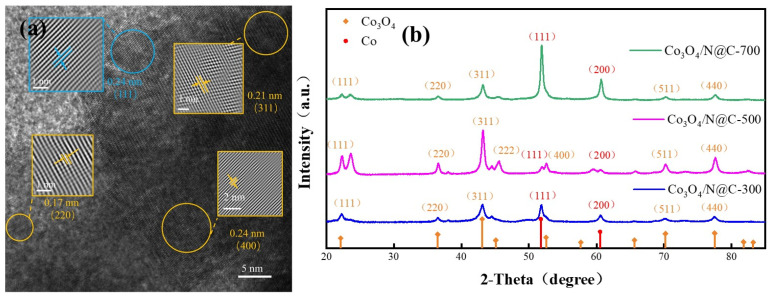
(**a**) TEM characterization of Co_3_O_4_/N@C-500; (**b**) XRD patterns of Co_3_O_4_/N@C-300, Co_3_O_4_/N@C-500, and Co_3_O_4_/N@C-700.

XRD analysis was employed to ascertain the crystalline phases in the Co_3_O_4_/N@C series synthesized at different carbonization temperatures (Figure 4). The Co_3_O_4_/N@C-300 sample exhibits characteristic peaks at 22.1°, 43.1° and 51.7°, assigned to metallic Co^0^ (111) (JCPDS#05-0727) and Co_3_O_4_ (311), (220) planes (JCPDS#42-1467), confirming co-existence of cobalt phases. For Co_3_O_4_/N@C-500, intensified peaks at 36.5° (220), 45.1° (222), 52.5° (400) and 74.1° (440) indicate enhanced Co_3_O_4_ crystallinity, while residual Co^0^ signatures persist at 51.8° (111) and 60.5° (200) (marked by red arrows). At 700 °C, the characteristic Co_3_O_4_ reflections dominate at 31.2° (220), 36.5° (311) and 59.4° (511) are accompanied by residual metallic cobalt signatures at 51.8° (111) and 60.5° (200), indicating partial oxidation under carbon-embedding conditions. TEM analysis of the representative Co_3_O_4_/N@C-500 (Figure 4a) corroborates these crystalline features, revealing distinct lattice spacings of 0.24 nm (Co^0^ (111)), 0.21 nm (Co_3_O_4_ (311)), and 0.17 nm (Co_3_O_4_ (400)), which align with XRD peak positions calculated. These structural findings further harmonize with SEM observations of homogeneous nanoparticles (Figure 2) and EDS elemental mapping demonstrating uniform spatial distribution of Co/O/N species without phase segregation.

**Figure 5:**
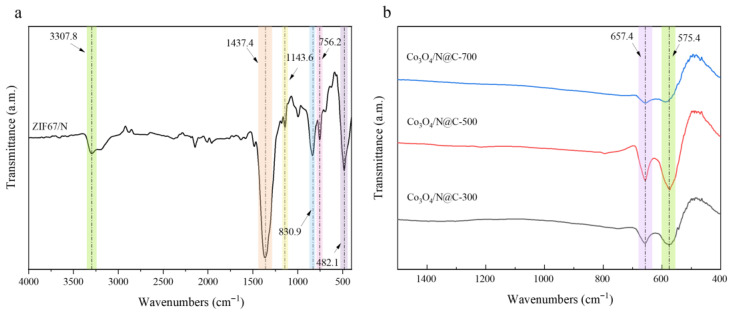
FTIR spectra of (**a**) ZIF-67/N; (**b**) Co_3_O_4_/N@C.

The functional groups of the four materials were detected with FT-IR spectroscopy. As depicted in Figure 5a, the pristine ZIF-67/N displays characteristic bands primarily attributed to the imidazole ring of 2-MI, with vibrational modes between 600 and 1500 cm^−1^ corresponding to ring structure stretching and deformation modes. Specifically, the prominent infrared signature at 756 cm^−1^ reflects combined C-N stretching and out-of-plane bending vibrations, while the 1144 cm^−1^ peak originates from in-plane bending vibrations. The spectral feature observed at 1421 cm^−1^ corresponds to concerted deformation and stretching modes of the imidazole heterocyclic framework. In particular, the peak at 756 cm^−1^ reflects combined C-N stretching and out-of-plane bending vibrations, while the 1144 cm^−1^ peak originates from in-plane bending vibrations. Additionally, the characteristic absorption band observed at 3308 cm^−1^ is attributable to O-H stretching vibrations [37]. Subsequent carbonization induces significant attenuation of imidazole-related peaks due to organic ligand decomposition. As evidenced by the FTIR spectra in Figure 5b, Co_3_O_4_/N@C exhibits characteristic metal-oxygen vibrations at 575 and 657 cm^−1^, corresponding to Co-O stretching modes. The vibrational band at 575 cm^−1^ is attributed to octahedrally coordinated Co^3+^ sites, whereas the absorption at 657 cm^−1^ corresponds to tetrahedral Co^2+^ moieties, collectively confirming the crystallization of spinel Co_3_O_4_ [38]. Among the synthesized catalysts, Co_3_O_4_/N@C-500 exhibits the most pronounced vibrational intensities at these characteristic wavenumbers, indicative of its superior cobalt-oxygen bond concentration.

**Figure 6:**
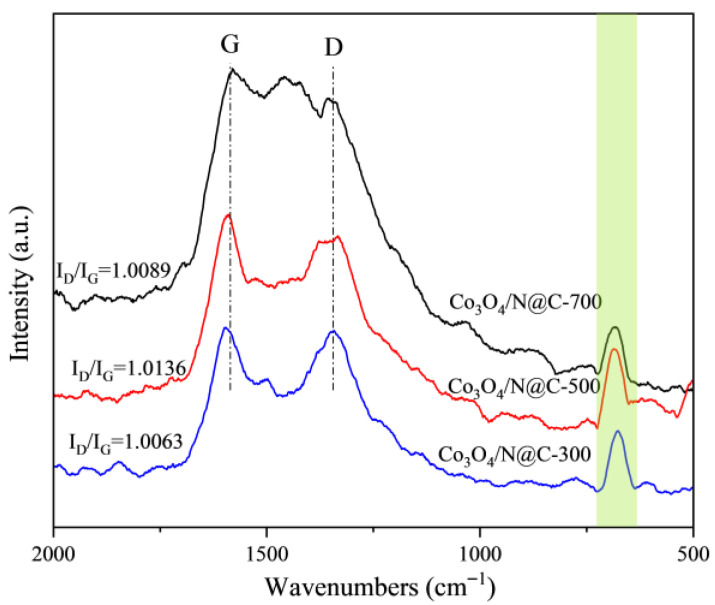
Raman analysis of Co_3_O_4_/N@C.

The Raman spectra of Co_3_O_4_/N@C materials (Figure 6) exhibit characteristic bands at 1340 cm^−1^ (D-band) and 1580 cm^−1^ (G-band) attributable to sp^3^-hybridized amorphous carbon domains and sp^2^-bonded graphitic lattices, respectively [39]. The intensity ratio ID/IG quantifies graphitization degree, with values of 1.0063 (Co_3_O_4_/N@C-300), 1.0136 (Co_3_O_4_/N@C-500), and 1.0089 (Co_3_O_4_/N@C-700). These near-unity ratios reflect substantial graphitic carbon content in all samples. Co_3_O_4_/N@C-500 demonstrates the highest ID/IG value, indicating its optimal graphitization for enhancing PDS activation via electron transfer. A weak peak at 688 cm^−1^ corresponds to the A_1_g vibrational mode of Co_3_O_4_, consistent with XRD data [40]. Notably, Raman signatures of cobalt nanocrystals were undetected due to doping levels below the instrumental detection limit.

**Figure 7:**
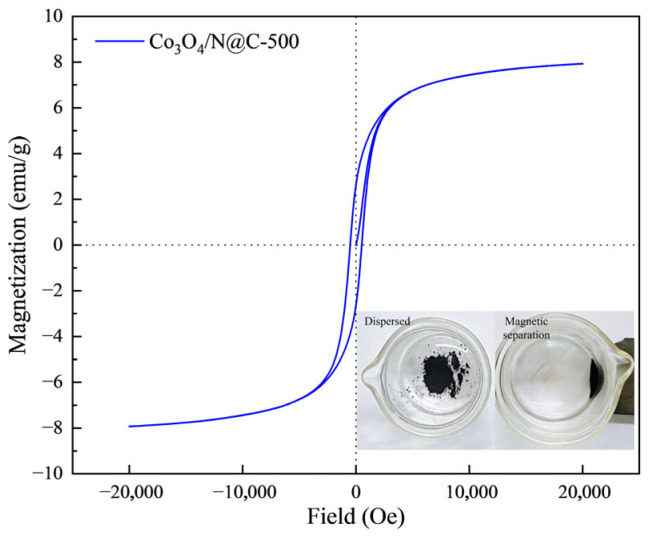
VSM characterization of Co_3_O_4_/N@C-500.

The hysteresis loop test of Co_3_O_4_/N@C-500 shows that the material exhibits superparamagnetic properties. The saturation magnetization of Co_3_O_4_/N@C-500 is about 7.9 emu/g in a 20,000 G magnetic field. Under an external magnetic field, the Co_3_O_4_/N@C-500 catalyst can be easily separated from the solution system, which improves the recovery efficiency of the catalyst and facilitates the recycling of the catalyst, as shown in Figure 7.

**Figure 8:**
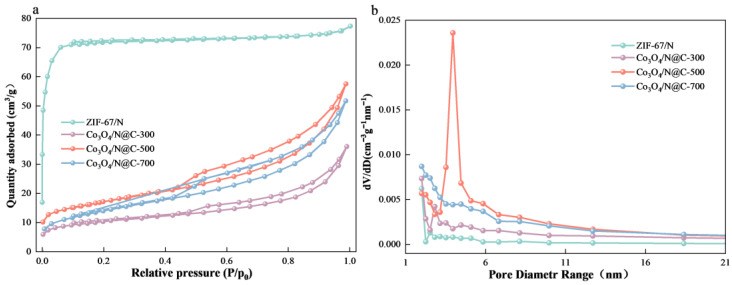
(**a**) Nitrogen adsorption–desorption isotherm; (**b**) pore size distribution.

The N_2_ adsorption–desorption analysis was performed to quantify accessible surface areas and pore architectures (Figure 8). The N_2_ adsorption–desorption isotherms of all Cu- Co_3_O_4_/N@C-X samples exhibit a characteristic type II profile, which is indicative of reversible multilayer adsorption on the material surface. Additionally, the presence of a distinct H3-type hysteresis loop in the desorption branch suggests the existence of a mesoporous structure within the Co_3_O_4_/N@C-X materials. Pyrolysis induces pervasive structural collapse in all derivatives, evidenced by drastic surface area reductions versus microporous ZIF-67/N (277.5 → 36.4 m^2^/g at 300 °C, 60.2 m^2^/g at 500 °C, 50.6 m^2^/g at 700 °C) (Table 1). Nevertheless, Co_3_O_4_/N@C-500 exhibits optimally balanced porosity: its distinct hysteresis loop in Figure 8a (P/P_0_ = 0.45–0.95) confirms interconnected mesopores, while the dominant 3–6 nm pore distribution (79% volume share, Figure 8b peak) exceeds antibiotic molecular dimensions (~1.5 nm), enabling accelerated reactant diffusion. This synergy of retained microporosity and tailored mesopores yields a 19.2% higher surface area than the 700 °C material (60.2 vs. 50.5 cm^3^/g). Notably, Co_3_O_4_/N@C-500 also possesses the largest pore volume among the series, underscoring its promise as a superior catalyst.

**Figure 9:**
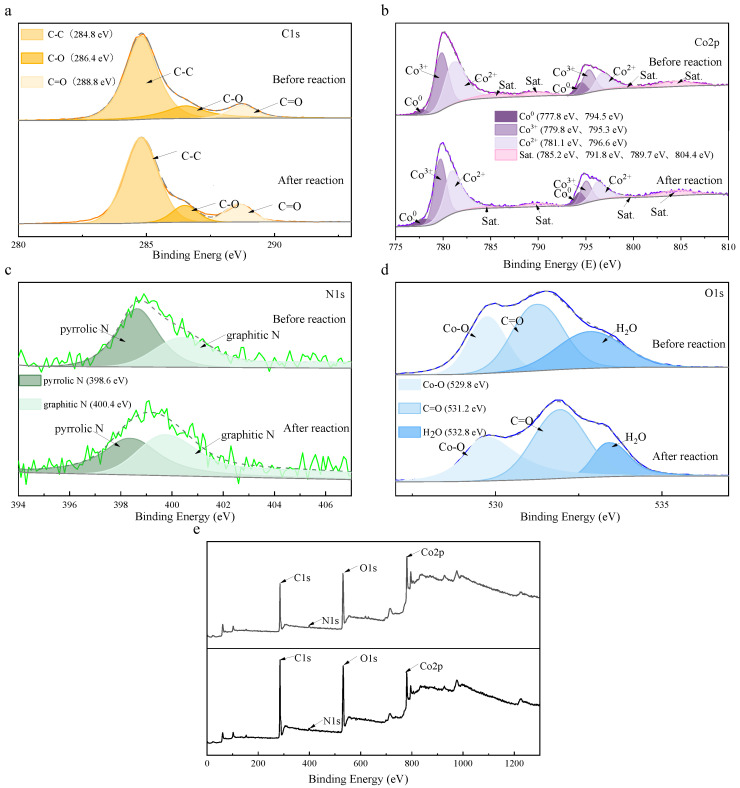
Co_3_O_4_/N@C-500 XPS. (**a**) C1s; (**b**) Co2p; (**c**) N1s; (**d**) O1s; (**e**) survey before and after the reaction.

XPS analysis was employed to probe the elemental composition, chemical states, and catalytic mechanisms of the Co_3_O_4_/N@C-500 surface (Figure 9). The full survey spectrum (Figure 9e) exhibits distinct signals for cobalt, oxygen, nitrogen, and carbon, consistent with EDS elemental mapping results (Figure 3), confirming material stability following PDS activation. High-resolution C1s spectra (Figure 9a) reveal three characteristic binding energies: 284.8 eV corresponding to C-C bonds, 286.4 eV to C-O bonds, and 288.8 eV to C=O bonds, primarily derived from graphitic carbon frameworks and residual organic ligands of the metal–organic framework precursor [41,42]. Analysis of the Co 2p region (Figure 9b) identifies coexisting cobalt oxidation states through peaks at 779.8 eV and 795.3 eV assigned to Co^3+^ species [43], alongside 781.1 eV and 796.6 eV signatures characteristic of Co^2+^ cations [44], with minor contributions from Co^0^ (777.8 eV and 794.5 eV) [45]. The N 1s spectrum (Figure 9c) demonstrates nitrogen incorporation via pyridinic nitrogen (398.6 eV) and graphitic nitrogen (400.4 eV), both enhancing electron transfer capabilities. Oxygen species analysis (Figure 9d) resolves contributions at 529.8 eV (Co-O bonds), 531.2 eV (surface hydroxyl groups), and 532.8 eV (C-O moieties) [46], collectively facilitating radical generation pathways. The synergistic interplay between multivalent cobalt centers and nitrogen-doped carbon matrices optimizes PDS activation through coupled electron transfer and redox cycling mechanisms.

### 3.2. Catalytic Performance

Firstly, the adsorption of CIP solution with different initial concentrations was studied, as shown in Figure S1. The activity of the catalytic material is affected by its own adsorption performance. Good adsorption performance can make the substances involved in the reaction quickly gather to the active center on the surface of the catalyst, improve the mass transfer efficiency in the reaction process, and effectively improve the catalytic reaction rate. At the same time, it can also eliminate the interference of adsorption and truly reflect the contribution of material catalytic performance to the reaction process. In different initial concentrations of CIP solution, all test materials can quickly reach the adsorption equilibrium or slow adsorption stage within 30 min. At an initial CIP concentration of 5 mg/L, the three materials achieved maximum CIP adsorption efficiencies of 35%, 60%, and 65%, respectively. As the initial concentration increases, the adsorption amount also increases.

Elevating initial CIP concentration to 10 mg/L yielded equilibrium adsorption capacities of approximately 3.0, 4.0, and 5.0 mg/g for the three materials, respectively. Upon further elevation to 20 mg/L, the corresponding adsorption capacities increased to 3.6 mg/g, 4.3 mg/g, and 6.8 mg/g. These concentration-dependent adsorption profiles collectively demonstrate that adsorption performance is positively correlated with initial contaminant loading, yet complete CIP removal remains unattainable due to saturation limitations inherent to the adsorption process.

The textural properties presented in Table 1 reveal that the catalyst pyrolyzed at 500 °C possesses the largest specific surface area and pore volume. In addition, a relatively uniform pore size distribution centered around 3.9 nm. These structural characteristics are critical for the adsorption process, as a high surface area provides more active sites [47], while a suitable pore size facilitates the diffusion and transport of CIP molecules to the active centers [48]. The well-developed porosity contributes significantly to the enhanced adsorption capacity, thereby promoting the subsequent catalytic degradation efficiency [49].

**Figure 10:**
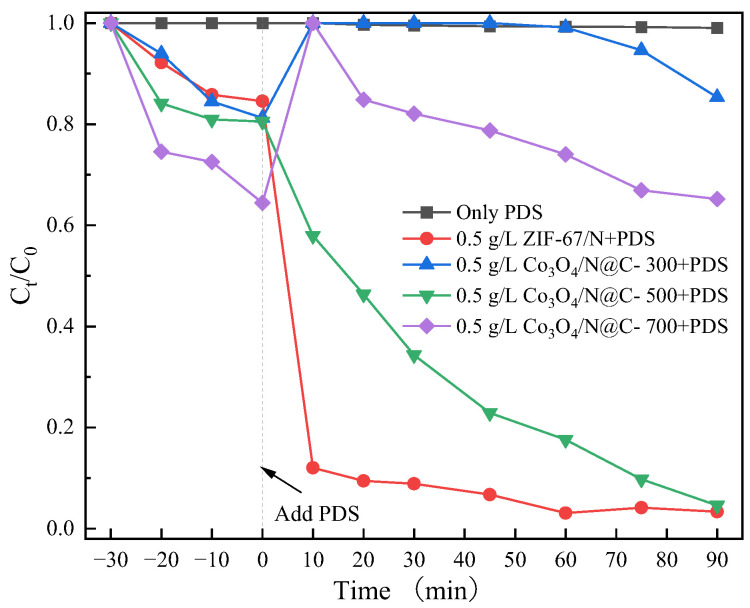
Degradation performance of different catalysts. Initial experiment conditions: [catalyst]_0_ = 0.5 g/L, [PDS]_0_ = 2 mmol/L, [CIP]_0_ = 20 mg/L, pH_0_ = 3.5, T = 25 °C.

The catalytic performance of four distinct catalysts (ZIF-67/N, Co_3_O_4_/N@C-300, Co_3_O_4_/N@C-500, and Co_3_O_4_/N@C-700) was systematically evaluated through PDS activation for CIP degradation in aqueous solution (Figure 10). Under standardized conditions (initial CIP = 20 mg/L, catalyst dosage = 0.5 g/L, PDS = 2 mmol/L), PDS alone exhibited negligible degradation (C/C_0_ ≈ 1.0). Significantly enhanced degradation was observed with ZIF-67/N and Co_3_O_4_/N@C-500, achieving 90 min CIP removal efficiencies of 97% and 96%, respectively. By contrast, Co_3_O_4_/N@C-300 and Co_3_O_4_/N@C-700 showed limited efficiencies of 15% and 35%. Although ZIF-67/N demonstrated rapid initial activation kinetics, its practical application was constrained by progressive cobalt leaching, leading to irreversible active site loss and poor reusability (Table S1). Conversely, Co_3_O_4_/N@C-500 displayed superior stability and environmental adaptability, indicating strong potential for scale-up implementation.

This distinct performance divergence stems primarily from the carbonization temperature-dependent structural evolution. Catalytic efficiency progressively improved as temperature increased from 300 °C to 500 °C, with Co_3_O_4_/N@C-500 achieving optimal activity through synergistic effects: enhanced carbon matrix graphitization facilitating electron transfer [50,51], development of larger specific surface area (60.2 m^2^/g), and appropriate dispersion of cobalt-active species. These structural advantages collectively promoted PDS activation to generate radicals, thereby accelerating CIP degradation kinetics.

However, exceeding the optimal temperature threshold induced significant structural deterioration. When carbonization reached 700 °C, the catalytic activity of Co_3_O_4_/N@C-700 drastically declined due to interconnected factors: excessive aggregation of Co_3_O_4_ nanoparticles causing active site reduction, pore collapse from carbon skeleton over-graphitization, and consequent impairment of reactant mass transfer. This multifactorial degradation ultimately compromised radical generation efficiency and surface accessibility of active sites. Table S2 shows the comparison of Co_3_O_4_/N@C-500 catalyst with other ZIF-67 derived materials or carbon-based materials. The experimental results show that the catalyst has a very significant effect on the degradation of CIP by catalytic PDS, indicating that the catalyst is also an excellent catalyst.

**Figure 11:**
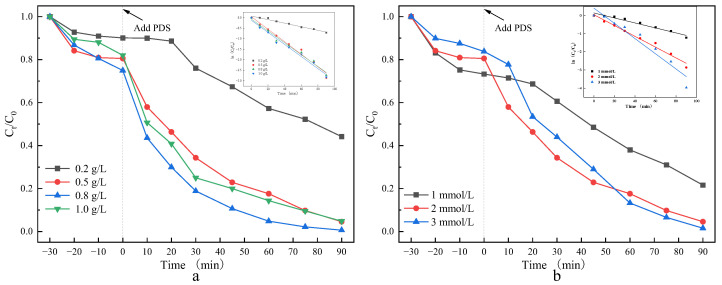
(**a**) Co_3_O_4_/N@C-500 dosage; (**b**) Reaction conditions PDS dosage on CIP degradation by Co_3_O_4_/N@C-500. Except for the studied condition, the others were fixed on [catalyst]_0_ = 0.5 g/L, [PDS]_0_ = 2 mmol/L, [CIP]_0_ = 20 mg/L, pH_0_ = 3.5, T = 25 °C.

**Figure 12:**
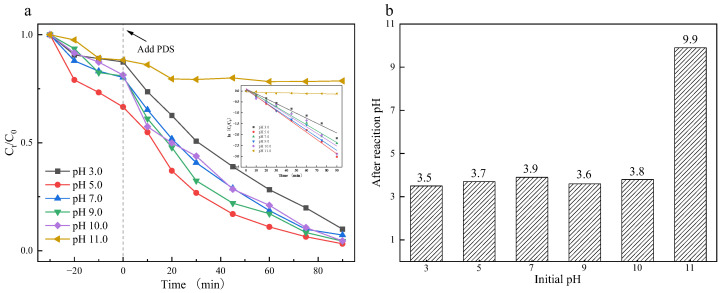
(**a**) Initial pH on CIP degradation by Co_3_O_4_/N@C-500; (**b**) The pH of the solution after reaction. Except for the studied condition, the others were fixed on [catalyst]_0_ = 0.5 g/L, [PDS]_0_ = 2 mmol/L, [CIP]_0_ = 20 mg/L, T = 25 °C.

The oxidation efficiency of the Co_3_O_4_/N@C-500/PDS system was investigated by evaluating the influence of catalyst dosage and PDS concentration on CIP degradation (Figure 11). Figure 12a demonstrates the catalyst dosage effect (0.2–1.0 g/L) on CIP elimination. Degradation efficiency initially increased from 55% to a maximum of 99.35% at 0.5 g/L, then declined to 95.16% at 1.0 g/L. Correspondingly, the pseudo-first-order rate constant (*k*_obs_) rose from 0.00846 min^−1^ to 0.02976 min^−1^ at 0.5 g/L, before decreasing to 0.02873 min^−1^ at 1.0 g/L (Table S3). The optimal performance at 0.5 g/L is attributed to sufficient active site availability for PDS activation and ROS generation. Beyond this threshold, catalyst agglomeration occurred, partially shielding active sites and reducing activation efficiency.

Figure 12b reveals the impact of PDS concentration (1–3 mmol/L). Degradation efficiencies reached 78%, 95%, and 98% at 1, 2, and 3 mmol/L, respectively. The rate constant (*k*_obs_) peaked at 0.02976 min^−1^ with 2 mmol/L PDS (Table S4). Although efficiency proportionally increased with oxidant dosage, the marginal improvement beyond 2 mmol/L indicates nonproductive oxidant consumption through self-quenching reactions. Considering both degradation kinetics and economic viability, 0.5 g/L catalyst and 2 mmol/L PDS were selected as optimal parameters.

CIP degradation efficiency was assessed across pH 3.0–11.0 to quantify pH-mediated impacts on reaction kinetics. Figure 12 reveals suppressed CIP degradation is hindered under strong alkalinity conditions. The degradation efficiency of CIP was significantly lower at pH 11 (21.4%) compared to rates exceeding 90% observed within the pH range of 3.0 to 10.0. Maximum degradation efficiency, indicated by a peak rate constant of 0.3311 min^−1^, occurred at pH 5 (Table S5). The reduced efficiency under strong alkaline conditions (pH 11) stems from the reaction between SO_4_·^−^ and hydroxide ions, lowering the reaction rate [52]. Given that the pH of most actual wastewater ranges from 5 to 9, the Co_3_O_4_/N@C-500 system shows strong potential for practical organic pollutant degradation.

In addition, the pH of the solution after the catalytic reaction was also detected. Within the experimentally controlled initial pH range (3–11), the pH of the solution after the reaction remained in the acidic range, which were 3.5, 3.7, 3.9, 3.6, 3.8 and 9.9, respectively (Figure 12b), indicating that Co_3_O_4_/N@C-500 has a certain buffer capacity for the pH of the solution during the reaction [20], so that the catalyst has a wide pH range. Cobalt ion leaching experiments demonstrated that the degradation efficiency of CIP remained above 90% across pH 3–10, while cobalt ion leaching decreased from 29.60 mg/L to 0.61 mg/L (Table S1).

### 3.3. Reusability of Co_3_O_4_/N@C-500

**Figure 13:**
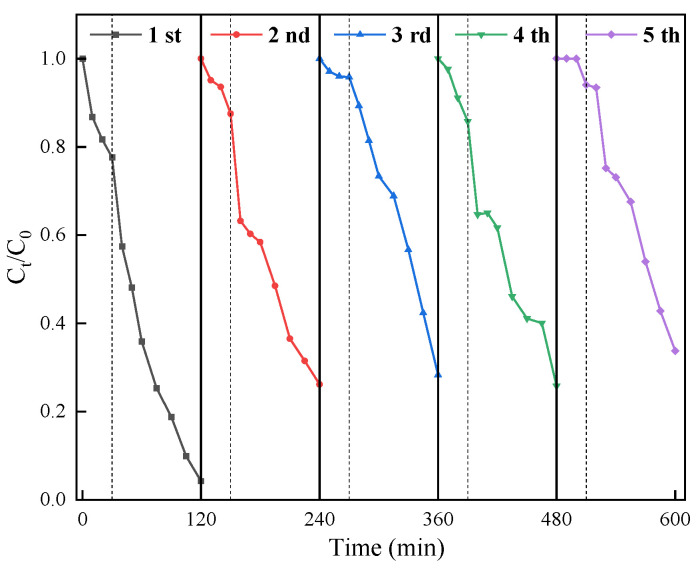
The reusability of Co_3_O_4_/N@C-500. Initial experiment conditions: [catalyst]_0_ = 0.5 g/L, [PDS]_0_ = 2 mmol/L, [CIP]_0_ = 20 mg/L, pH_0_ = 5, T = 25 °C.

To evaluate the catalyst’s stability and reusability, it was subjected to consecutive degradation cycles (Figure 13). Following each reaction cycle, the catalyst was isolated via filtration, rinsed copiously with deionized water, and subsequently desiccated in a forced-air oven. The collected catalyst was then reused in subsequent degradation assays conducted under the consistent experimental parameters. During the initial cycle, a CIP degradation efficiency of 97.88% was achieved within 90 min. However, upon the first reuse, the degradation efficiency decreased significantly to 73.82%. A further slight reduction to 71.69% was observed after the second reuse. Similar situations have also been approved in other relevant studies [53]. The primary factor contributing to the attenuation of CIP degradation efficiency may be that CIP and its degradation intermediates/by-products accumulate on Co_3_O_4_/N@C-500, and the adsorption performance of the material decreases. Simultaneously, the active sites exposed on the surface of the material are reduced, and the passivation phenomenon occurs. As a result, the activity of the catalyst is gradually attenuated. To maintain the activity of the catalyst, the used Co_3_O_4_/N@C-500 underwent thermal treatment at 300 °C for 1 h in a N_2_ atmosphere [54]. The catalytic degradation of the material increased to 74.2% after thermal activation. Subsequently, the degradation performance of CIP was maintained at 66.2%.

**Figure 14:**
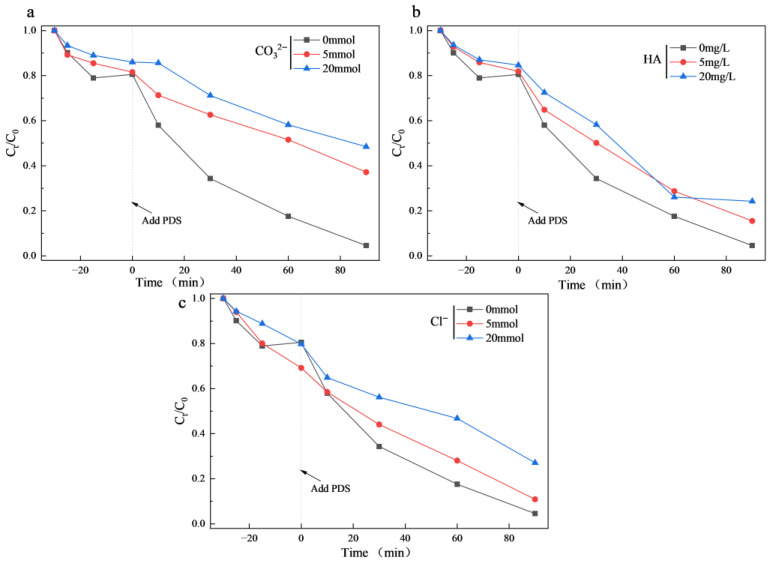
Influence of co-existing substances on the degradation performance of the Co_3_O_4_/N@C-500/PDS/CIP system: (**a**) CO_3_^2−^, (**b**) HA, and (**c**) Cl^−^. (Initial experiment conditions: [catalyst]_0_ = 0.5 g/L, [PDS]_0_ = 2 mmol/L, [CIP]_0_ = 20 mg/L, pH_0_ = 5, T = 25 °C.

In addition, it is required to investigate the effects of these common substances on CIP degradation. As shown in Figure 14, CO_3_^2−^, Cl^−^ and humic acid (HA) exhibited inhibitory effects on the reaction to varying degrees. Among them, CO_3_^2−^ showed the most significant inhibition: when its concentration was increased to 5 mmol and 20 mmol, the degradation rate of CIP decreased to 58% and 74.4% at 120 min, respectively. This can likely be attributed to the complexation between carbonate ions and metal active sites, which reduced the number of available active sites and thus decreased the catalytic activity [55]. In comparison, Cl^−^ exhibited a relatively weaker inhibitory effect on CIP degradation. Furthermore, HA also caused certain suppression of the degradation. At an HA concentration of 5 mg/L, the CIP degradation rate was 88.8%. When the HA concentration was increased to 20 mg/L, the degradation efficiency further decreased. This may be due to competitive adsorption between HA and CIP on the active sites, consuming some of the reactive species. In summary, both anions and humic acid showed certain inhibitory effects on CIP degradation, though the overall extent of inhibition remained relatively limited.

In order to evaluate the universality of Co_3_O_4_/N@C-500, two different types of organic pollutants, including sulfadiazine (SDZ) and tetracycline hydrochloride (TCH), were selected, as shown in Figure S2. The degradation efficiencies of SDZ and TCH by Co_3_O_4_/N@C-500/PDS system were 88.5% and 85.6% within 120 min, respectively. In addition, the performance of the catalyst in tap water was tested to study the effect of water quality on CIP degradation. The degradation efficiency of CIP was 84.3% within 120 min. The results show that Co_3_O_4_/N@C-500 has s good stability and practical application potential (Figure S3).

### 3.4. Identification of Reactive Oxide Species and Possible Catalytic Mechanism

#### 3.4.1. Recognition of ROS

**Figure 15:**
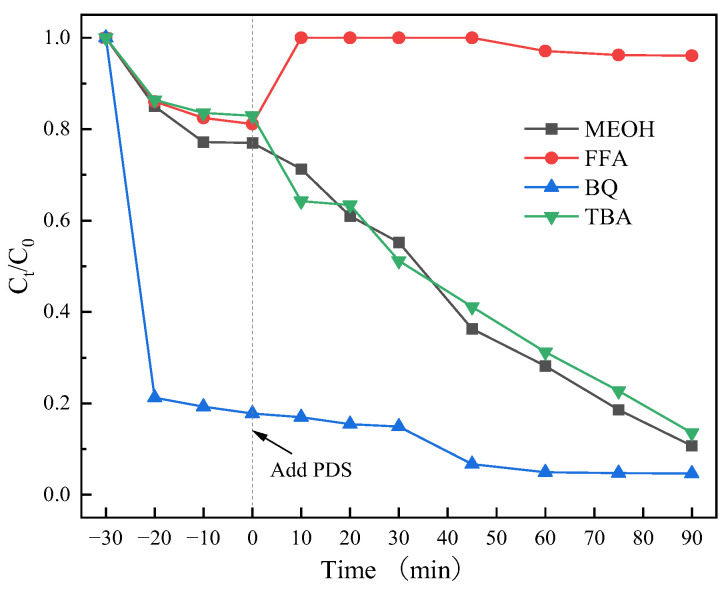
Influences of different scavengers on CIP degradation over Co_3_O_4_/N@C-500. Initial experiment conditions: [catalyst]_0_ = 0.5 g/L, [PDS]_0_ = 2 mmol/L, [CIP]_0_ = 20 mg/L, pH_0_ = 5, T = 25 °C.

To delineate the distinct contributions of radical and non-radical pathways to CIP degradation, radical scavenging assays were conducted with selective quenchers (Figure 15). MeOH can simultaneously quench SO_4_·^−^ and ·OH (*k*_SO4·_^−^ = 1.1 × 10^7^ M^−1^s^−1^, *k*_·OH_ = 9.7 × 10^8^ M^−1^s^−1^), tert-butanol can only effectively quench ·OH (*k*_·OH_ = 6 × 10^8^ M^−1^s^−1^), BQ can quickly react with superoxide anion radical (O_2_·^−^) in the system (*k*_O2·_^−^ = 5.0 × 10^8^ M^−1^s^−1^), FFA is usually used to quench ^1^O_2_ in the system (*k*^1^_O2_ = 5.0 × 10^8^ M^−1^s^−1^) [20,56]. The ratio of quenching agent and PDS was 50:1. After adding BQ, the degradation efficiency of CIP was 95.35%. Compared with the degradation efficiency without quencher (95.41%), the degradation of CIP was almost unaffected, indicating that O_2_·^−^ in the Co_3_O_4_/N@C-500/PDS catalytic system was not an active oxygen substance involved in the degradation of CIP.

When TBA was added to the CIP degradation reaction system, the degradation efficiency was 86.45%. In contrast, the addition of MeOH resulted in a degradation efficiency of 89.28%. However, the addition of FFA significantly inhibited CIP degradation, yielding an efficiency of only 3.92%. Collectively, these results demonstrate the non-participation of SO_4_*·*^−^ radicals in CIP degradation, and ·OH are not the primary reactive species. Instead, ^1^O_2_ plays the major role in degrading CIP.

**Figure 16:**
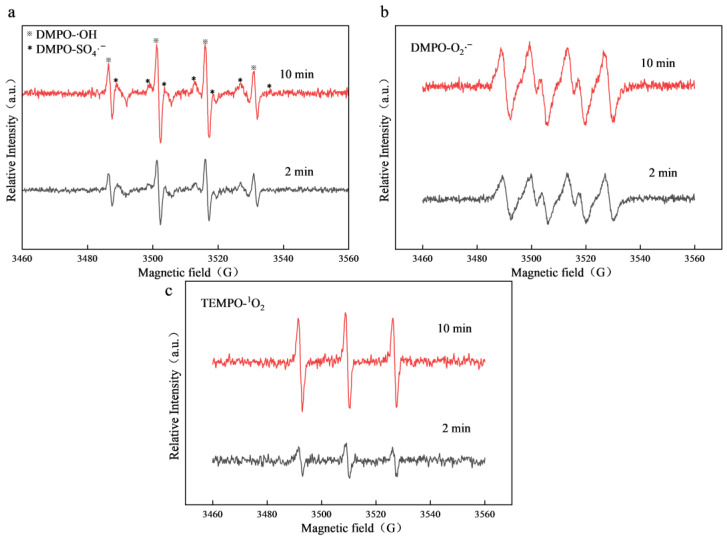
(**a**) Measured EPR spectra using DMPO-·OH; (**b**) EPR spectra of DMPO-O_2_·^−^; and (**c**) EPR spectra of TEMPO-^1^O_2_.

In order to further confirm the conclusion of the above free radical quenching experiment, the free radicals produced in the Co_3_O_4_/N@C-500/PDS system were captured and identified by EPR. SO_4_·^−^ and ·OH in the system can be captured by DMPO as a radical scavenger in pure water [57,58]. EPR analysis of the Co_3_O_4_/N@C-500/PDS system revealed distinct signals for both DMPO-·OH and DMPO-SO_4_·^−^ adducts, with the latter exhibiting significantly lower intensity. This attenuated SO_4_·^−^ signal suggests potential conversion of SO_4_·^−^ to ·OH during the catalytic process. With the catalytic reaction, the ·OH signal is enhanced, indicating that the system can continuously produce ·OH and has a certain accumulation (Figure 16a). Using MeOH as a solvent to quench SO_4_·^−^ and ·OH, DMPO as a free radical scavenger, four strong and two weak six-fold peaks of DMPO-O_2_·^−^ can be observed [57,58], as shown in Figure 16b, O_2_·^−^ can be continuously generated in the system as the reaction proceeds. With TEMPO as the capture agent, a triple characteristic peak of TEMPO-^1^O_2_ peak was detected in the catalytic system, as shown in Figure 16c. The ^1^O_2_ in the system also increased with the continuous signal of the reaction. Although ·OH, SO_4_·^−^ and O_2_·^−^ were detected by EPR, quenching experiments and kinetic analysis indicate that their contributions to CIP degradation are minimal compared to the non-radical pathway dominated by ^1^O_2_. Therefore, the primary mechanism for PDS activation by Co_3_O_4_/N@C-500 can be attributed to a non-radical pathway involving ^1^O_2_. The nature of the reactive species also implies a specific degradation profile for CIP.

#### 3.4.2. Catalytic Mechanism

**Figure 17:**
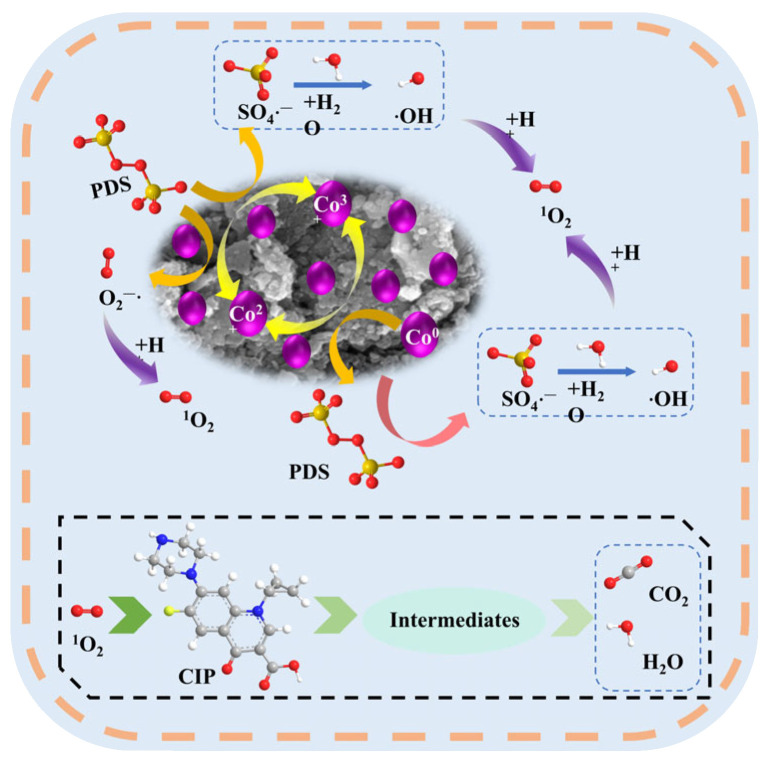
The possible catalytic mechanism of the Co_3_O_4_/N@C-500/PDS process.

Combined with the analysis, the Co_3_O_4_/N@C-500/PDS system mediated CIP degradation primarily through non-radical mechanisms (Figure 17). XPS analysis of catalyst surfaces before and after use (Figure 9) revealed dynamic valence changes: cobalt speciation shifted with Co^3+^ proportion decreasing from 46.2% to 41.4%, while Co^0^ and Co^2+^ increased from 6.78% to 9.08% and 47.04% to 49.5%, respectively (Figure 9b), confirming redox cycling during catalysis. Concurrently, nitrogen speciation analysis (Figure 9c) demonstrated decreased graphitic nitrogen alongside increased pyridinic nitrogen content, indicating participation of both nitrogen types in PDS activation. Electron transfer within the system was facilitated by carbon-oxygen bonds possessing high redox potential, while hydroxyl groups served dual roles as electron-donating moieties and active sites. These components synergistically enhanced PDS activation for contaminant degradation [45,59].

The catalytic mechanism involves synergistic redox cycles mediated by cobalt species and nitrogen functionalities [60]. Metallic cobalt (Co^0^) activates PDS to generate SO_4_·^−^ while oxidizing to Co^2+^ (Equation (1)). Subsequently, Co^2+^ further activates PDS through Equations (2) and (3), producing SO_4_·^−^ and ·OH while oxidizing to Co^3+^. The resultant Co^3+^ undergoes reduction back to Co^2+^ via PDS consumption (Equation (4)), establishing a closed-loop redox cycle. Concurrently, Co^0^ accelerates this cycle by directly reducing Co^3+^ to Co^2+^ (Equation (5)). This Co^0^/Co^2+^/Co^3+^ interconversion sustains efficient PDS activation for CIP degradation. Pyridinic and graphitic nitrogen dopants enhance electron transfer between the carbon matrix and PDS, optimizing oxidant activation efficiency. ^1^O_2_ generation occurs through dual pathways: direct PDS self-decomposition and radical-mediated reactions where superoxide (O_2_·^−^) reacts with ·OH/OH^−^ (Equations (6) and (7)).

Co^0^ + 2S_2_O_8_^2−^ → Co^2+^ + 2SO_4_^2−^ + 2SO_4_·^−^ (1)

Co^2+^ + S_2_O_8_^2−^ → Co^3+^ + SO_4_^2−^ + SO_4_·^−^ (2)

SO_4_·^−^ + H_2_O → SO_4_^2−^ +·OH + H^+^ (3)

Co^3+^ + 2H_2_O + S_2_O_8_^2−^ → Co^2+^ + 2SO_4_^2−^ + O_2_·^−^ + 4H^+^ (4)

2Co^3+^ + Co^0^ → 3Co^2+^ (5)

·OH + O_2_·^−^ → ^1^O_2_ + OH^−^ (6)

2O_2_·^−^ + 2H^+^ → ^1^O_2_ + H_2_O_2_ (7)

## 4. Conclusions

In this study, the ZIF-67/N was prepared at room temperature by adding ammonia, which was nearly four times the yield of ZIF-67 without ammonia. Subsequently, a nitrogen-doped carbon composite, denoted as Co_3_O_4_/N@C, was obtained through carbonization in a nitrogen atmosphere. The successful incorporation of C, O, N, and Co onto the catalyst surface was confirmed by a series of characterization techniques, including SEM, TEM, XRD, and XPS. Evaluation under different calcination temperatures revealed that Co_3_O_4_/N@C-500 exhibited the optimal performance. Comprehensive SEM, HRTEM, EDS, and XRD revealed that Co_3_O_4_ is uniformly dispersed in the carbon matrix. Such a structure significantly enhances the material’s activity and stability. Under ideal reaction conditions, this catalyst demonstrated a CIP degradation efficiency of 95%, highlighting its superior catalytic properties. Repeated experiments confirmed the notable stability of the catalyst, as it maintained high degradation efficiency over five successive cycles. The mechanism study shows that ·OH, SO_4_·^−^, O_2_·^−^, and ^1^O_2_ appear in the system, with ^1^O_2_ playing the dominant role. By engineering multifunctional MOF materials, this work provides a scalable technological framework for the efficient degradation of recalcitrant organic contaminants in complex wastewater matrices.

## Figures and Tables

**Table 1:** Texture properties of the materials.

Sample	BET Surface Area (m^2^/g)	Pore Volume (cm^3^/g)	Average Pore Size (nm)
ZIF-67/N	277.5	0.119	1.7
Co_3_O_4_/N@C-300	36.4	0.056	6.1
Co_3_O_4_/N@C-500	60.2	0.089	5.9
Co_3_O_4_/N@C-700	50.5	0.080	6.3

## Data Availability

The original contributions presented in this study are included in the article/Supplementary Material. Further inquiries can be directed to the corresponding authors.

## Supplementary Materials

The following supporting information can be downloaded at: https://www.mdpi.com/article/10.3390/ma18184337/s1, Figure S1. Different materials for different initial concentrations of CIP Adsorption performance. (a) 5 mg/L; (b) 10 mg/L; (c) 20 mg/L. Initial experiment conditions: [catalyst]_0_=0.5 g/L, [CIP]_0_=20 mg/L, pH_0_=3.5, T=25 °C. Figure S2. Effects of degradation of different pollutants by Co_3_O_4_/N@C-500. Initial experiment conditions: [catalyst]_0_=0.5 g/L, [CIP]_0_=20 mg/L, pH_0_=3.5, T=25 °C. Figure S3. Effects of degradation of different pollutants and real water matrix by Co_3_O_4_/N@C-500. Initial experiment conditions: [catalyst]_0_=0.5 g/L, [CIP]_0_=20 mg/L, pH_0_=3.5, T=25 °C. Table S1. Co ion dissolution of different catalysts under different initial pH condition. Table S2. The removal data of various catalysts in the PS activation system. Table S3. Parameters of pseudo-first-order kinetic model for the degradation of phenol with various Co_3_O_4_/N@C-500 dosage. Table S4. Parameters of pseudo-first-order kinetic model for the degradation of phenol with various PDS dosage. Table S5. Parameters of pseudo-first-order kinetic model for the degradation of phenol with various initial pH. References [61,62,63] have been cited in Supplementary file.
