# Supplementary material for: Ammonia-Assisted Quadrupled-Yield ZIF-67 Derivation Enables Single Oxygen-Dominated Nonradical Oxidation for Enhanced Ciprofloxacin Degradation
Source: Materials (Basel). 2025 Sep 16;18(18):4337. doi: 10.3390/ma18184337 (PMC12472137; doi:10.3390/ma18184337)
Supplement: Supplementary file 1 [file materials-18-04337-s001.zip › materials-3796549-supplementary.pdf]

Supplementary Materials

Ammonia-Assisted Quadrupled-Yield ZIF-67 Derivation Enables Single Oxygen-Dominated Nonradical Oxidation for Enhanced Ciprofloxacin Degradation

Xiaoxian Hu [1], Di Zhang [1,2], Xinyu Li [1,*], Junfeng Wu [1,*], Xiang Guo [1], Hongbin Gao [1], Minghui Hao [1], Yingchun Wang [1], Bang Li [1] and Xinhai Zhang [3]

1 Henan Key Laboratory of Water Pollution Control and Rehabilitation, Henan University of Urban Construction, Pingdingshan 467000, China; 20221057@huuc.edu.cn
2 College of Ecology and Environment, North China University of Water Resources and Electric Power, Zhengzhou 450046, China;
3 College of Food and Chemical Engineer, Hebi Polytechnic, Hebi 458030，China
* Authors to whom correspondence should be addressed

.

Text S1:

According to the experimental conditions in the manuscript, the cost of catalysts and oxidants for treating 1 mg of CIP is calculated as follows: the cost of catalysts is 0.109 $/mg, and the cost of oxidants is 0.001 $/mg. The costing estimated is based on laboratory prices for analytical reagents. In contrast, the cost of chemical reagents used in the large-scale production of catalysts are lower than laboratory cost estimates. The catalysts in this study have excellent recyclability and recoverability. Therefore, it can save the application cost of catalysts.

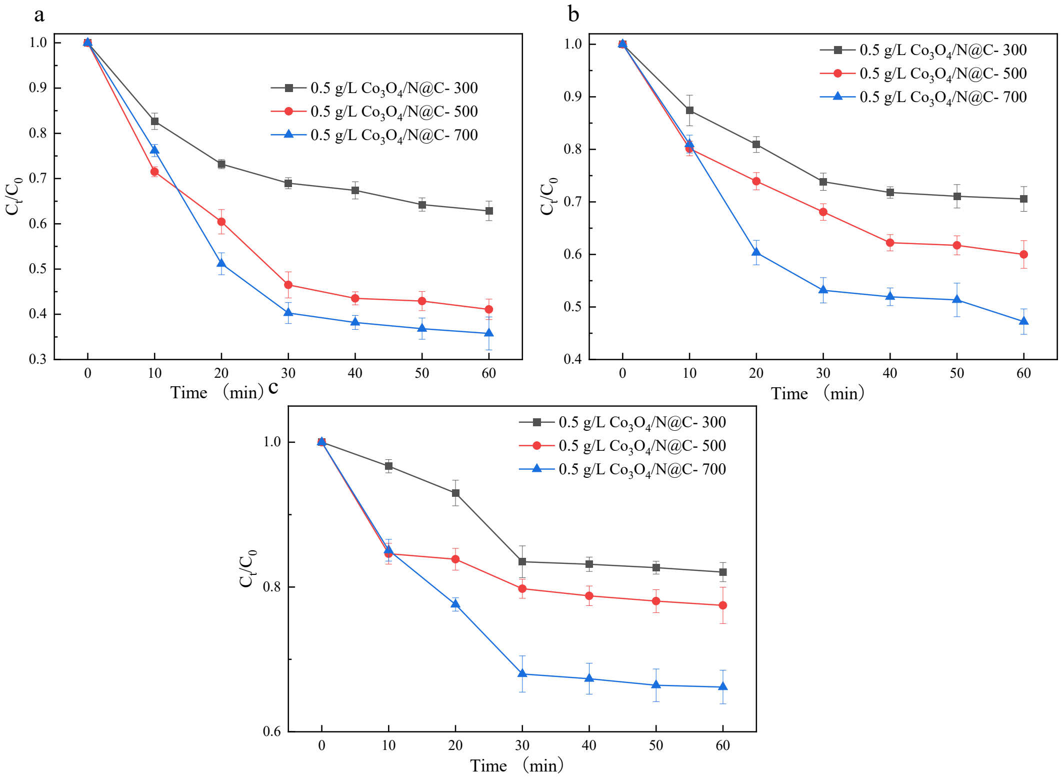

Figure S1. Different materials for different initial concentrations of CIP Adsorption performance. (a) 5 mg/L; (b) 10 mg/L; (c) 20 mg/L. Initial experiment conditions: $[catalyst]_0$=0.5 g/L, $[CIP]_0$=20 mg/L, pH_0=3.5, T=25 °C.

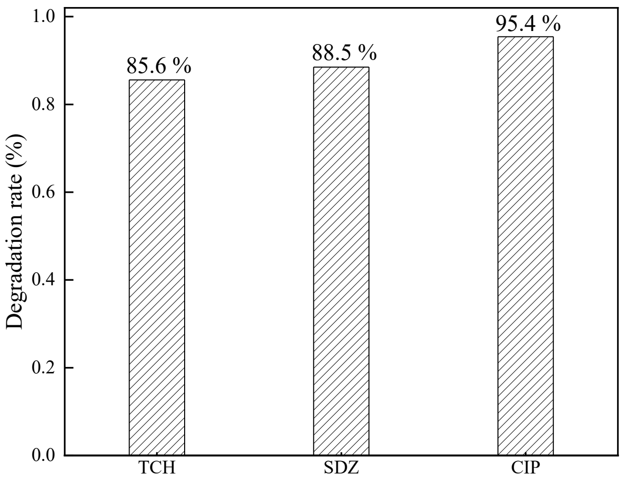

Figure S2. Effects of degradation of different pollutants by Co_3O_4/N@C-500. Initial experiment conditions: $[catalyst]_0$=0.5 g/L, $[CIP]_0$=20 mg/L, pH_0=3.5, T=25 °C.

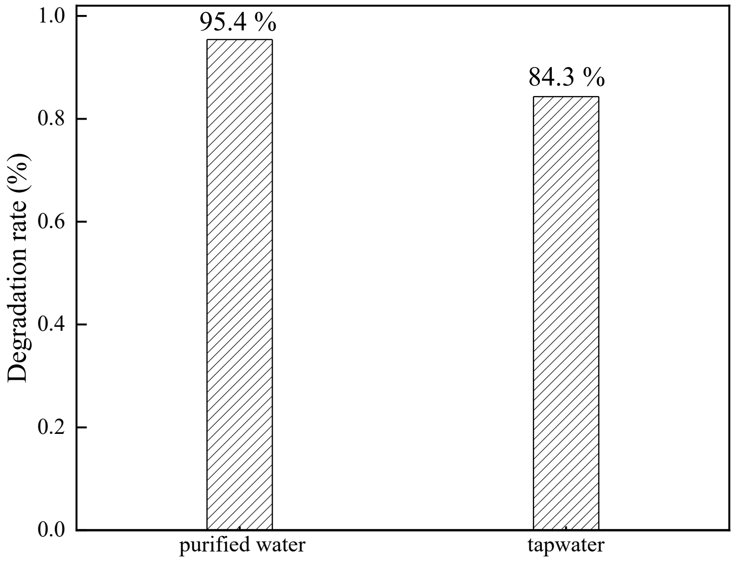

Figure S3. Effects of degradation of different pollutants and real water matrix by Co_3O_4/N@C-500. Initial experiment conditions: [catalyst]$_0$=0.5 g/L, [CIP]$_0$=20 mg/L, pH$_0$=3.5, T=25 °C.

Table S1. Co ion dissolution of different catalysts under different initial pH condition.

Catalyst	Co（mg/L）
ZIF-67/N, pH=3	38.4
Co_3O_4/N@C-500, pH=3	29.6
Co_3O_4/N@C-500, pH=5	7.17
Co_3O_4/N@C-500, pH=7	3.72
Co_3O_4/N@C-500, pH=9	5.73
Co_3O_4/N@C-500, pH=10	0.611
Co_3O_4/N@C-500, pH=11	0.062

Table S2. The removal data of various catalysts in the PS activation system.

Catalyst	oxidan	Optimal reaction conditions	Performance	Ref.
ZIF-67-300	PMS	[catalyst]=0.6 g/L, $[PMS]_0$=0.2 g/L, $[TC]_0$=20 mg/L	95% in 30 min	[1]
CoS_x/NF	PMS	[catalyst]=1 piece 2 × 2 cm NF, $[PMS]_0$=1 mM, $[OFL]_0$=20 mg/L	85% in 45 min	[2]
Co_3O_4/$NiCo_2O_4$ DSNCs	PDS	[catalyst]= 0.1 g/L, $[PDS]_0$= 74 μM, $[BPA]_0$=8 mg/L	76.7% in 18 min	[3]
1.0-BC/CoNC	PMS	[catalyst]= 0.2 g/L, $[PMS]_0$=1 mM, $[CIP]_0$=20 mg/L	95.9% in 30 min	[4]
CuO@ZIF-67(Co)	PMS	[catalyst]= 0.1 g/L, $[PMS]_0$= 0.05 g/L, $[MB]_0$=10 mg/L	100% in 1 min	[5]

Table S3. Parameters of pseudo-first-order kinetic model for the degradation of phenol with various Co_3O_4/N@C-500 dosage.

**Catalyst dosage (m/L)**	0.2	0.5	0.8	1.0
k_{obs}	0.008	0.030	0.028	0.028
**R^2**	0.971	0.928	0.985	0.983

Table S4. Parameters of pseudo-first-order kinetic model for the degradation of phenol with various PDS dosage.

**PDS dosage (mmol/L)**	1	2	3
k_{obs}	0.014	0.030	0.0419
R^2	0.962	0.982	0.935

Table S5. Parameters of pseudo-first-order kinetic model for the degradation of phenol with various initial pH.

**pH**	3	5	7	9	10	11
k_{obs}	0.023	0.033	0.027	0.031	0.029	0.001
**R^2**	0.971	0.994	0.991	0.986	0.9852	0.602

References

1. zhu, X.; Wang, T.; Sun, J.; Li, J.; Wang, J.; Wang, J.; Peng, R.; Li, Z.; Liu, J.; Jiang, L.; et al. Activation of peroxymonosulfate by ZIF-67-derived Co_3O_4 for the degradation of tetracycline: Effect of roasting temperature. *Inorganica Chimica Acta* **2024**, *568*.
2. Yang, Y.; Ye, J.; Zhai, Y.; Yang, B.; Yin, M.; Xu, Y.; Wang, J.; Zhang, X. ZIF-67-derived monolithic bimetallic sulfides as efficient persulfate activators for the degradation of ofloxacin J Surfaces and Interfaces. **2024**, *51*, 104713-104713.
3. Wang, M.; Cui, Y.; Cao, H.; Wei, P.; Chen, C.; Li, X.; Xu, J.; Sheng, G. Activating peroxydisulfate with Co_3O_4/$NiCo_2O_4$ double-shelled nanocages to selectively degrade bisphenol A; A nonradical oxidation process J Applied Catalysis B: Environmental. **2021**, *282*, 119585-.
4. Zhu, Z.; Qinqin, Y.; Youzhi, D.; Bo, F. Biochar supported magnetic ZIF-67 derivatives activated peroxymonosulfate for the degradation of ciprofloxacin: Radical and nonradical pathways J Colloids and Surfaces A: Physicochemical and Engineering Aspects. **2023**, *657*.
5. Xing, Y.; Fang, W.; Liang, Q.; Sun, M.; Lin, L.; Luo, H. A novel magnetic Fe, N co-doped Ce-MOFs derived carbon as a peroxymonosulfate activator for the degradation of tetracycline hydrochloride: Performance and activation mechanism J Journal of Water Process Engineering. **2024**, *60*, 105219-.
